# Identification of new progestogen-associated networks in mammalian ovulation using bioinformatics

**DOI:** 10.1186/s12918-018-0577-7

**Published:** 2018-04-03

**Authors:** Fang Yang, Meng Wang, Baoyun Zhang, Wei Xiang, Ke Zhang, Mingxin Chu, Pingqing Wang

**Affiliations:** 1 College of Bioengineering, Chongqing University, Chongqing, 400030 China; 2 Medical Molecular Biology Research Center, School of Basic Medical Sciences, Southwest Medical University, Luzhou, Sichuan, 646000 China; 3 Key Laboratory of Farm Animal Genetic Resources and Germplasm Innovation of Ministry of Agriculture, Institute of Animal Science, Chinese Academy of Agricultural Sciences, Beijing, 100193 China

**Keywords:** Progesterone, Bioinformatics, Progestogenic-associated, Mammalian ovulation

## Abstract

**Background:**

Progesterone plays an essential role in mammalian ovulation. Although much is known about this process, the gene networks involved in ovulation have yet to be established. When analyze the mechanisms of ovulation, we often need to determine key genes or pathways to investigate the reproduction features. However, traditional experimental methods have a number of limitations.

**Results:**

Data, in this study, were acquired from GSE41836 and GSE54584 which provided different samples. They were analyzed with the GEO2R and 546 differentially expressed genes were obtained from two data sets using bioinformatics (absolute log_2_ FC > 1, *P < 0.05*). This study identified four genes (*PGR*, *RELN*, *PDE10A* and *PLA2G4A*) by protein-protein interaction networks and pathway analysis, and their functional enrichments were associated with ovulation. Then, the top 25 statistical pathway enrichments related to hCG treatment were analyzed. Furthermore, gene network analysis identified certain interconnected genes and pathways involved in progestogenic mechanisms, including progesterone-mediated oocyte maturation, the MAPK signaling pathway, the GnRH signaling pathway and focal adhesion, etc. Moreover, we explored the four target gene pathways. q-PCR analysis following hCG and RU486 treatments confirmed the certain novel progestogenic-associated genes (*GNAI1*, *PRKCA*, *CAV1*, *EGFR*, *RHOA*, ZYX, *VCL*, *GRB2* and *RAP1A*).

**Conclusions:**

The results suggested four key genes, nine predicted genes and eight pathways to be involved in progestogenic networks. These networks provide important regulatory genes and signaling pathways which are involved in ovulation. This study provides a fundamental basis for subsequent functional studies to investigate the regulation of mammalian ovulation.

**Electronic supplementary material:**

The online version of this article (10.1186/s12918-018-0577-7) contains supplementary material, which is available to authorized users.

## Background

Mammalian reproduction depends on the perfect ovulation of a fully differentiated oocyte capable of fertilisation. This process is triggered by hormonal release at the hypothalamic-pituitary-ovarian axis [1, 2]. Ovulation is initiated by gonadotropin luteinising hormone (LH). This is a complex process that includes follicle-wall degradation, vasodilation, vascular dynamics and inflammation [2]. In this context, it is important to elucidate the mechanism of mammalian ovulation required for effective reproduction.

Progesterone and its receptor (progesterone receptor, PGR) are both required in the ovulation process. The inhibition of progesterone synthesis by mifepristone is known to block ovulation in rats [2]. The anovulatory phenotype of the *PGR*-null mouse with a distinct defect in follicle-wall breakdown presents an opportunity to investigate molecular pathways. It is known that PGR controls the expression of a number of downstream target genes which regulate the ovulatory process [2–5]. Studies have identified certain progesterone-related factors including estradiol (E2), epidermal growth factor (Egf) [6], prostaglandin-endoperoxide synthase 2 (Ptgs2) [7], vascular endothelial growth factor (Vegfa) [8] and interleukin-6 (Il6) [9]. Furthermore, experiments using *gene*-null mice identified unruptured follicles and the failure of follicle-wall breakdown as the two causes of impaired ovulation. This analysis revealed novel gene signaling pathways which control the activity of PGR downstream to regulate ovulation, including PGR-Hif-Adamts1 (follicle-wall degradation), the PGR-Hif-Vegfa (vascular permeability) and PGR-Pparγ-Il6-Ptgs2 (inflammation) pathways [2]. However, the process in which these systematic signaling networks are involved the regulation of ovulation has not been identified.

Unlike traditional methods, bioinformatics analysis can explore multi-component and multi-target molecular networks. In study of ovarian primordial follicle assembly, much of the information derived from sequencing data needs further analysis, including a systems biology approach to identify gene expression networks [10–15]. This approach can indicate novel factors and potential targets that correlate with ovarian disease. Grøndah et al. [16] described the transcriptome associated with oocyte development by in silico microarray analysis, identified differentially expressed genes by bioinformatics analysis, and further validated the identified genes through conducting quantitative real-time polymerase chain reactions (q-PCR) analysis and confirming the functional categories. These studies suggested that bioinformatics analysis based on sequencing data provided not only a systematic understanding of complex biological processes, but also a promising and efficient method to explore physiological functions in follicle development [13]. Current research focusing on the molecular pathways and networks involved in ovulation remains insufficient, and further bioinformatics analysis is needed.

The present study aims to explore gene networks and predict putative regulatory factors involved in ovulation by using bioinformatics analysis. Our objectives are to: 1) identify the most common and specific differentially expressed genes through investigating the protein-protein interactions; 2) determine the key candidate genes by constructing biological gene networks involved in progesterone production during ovulation; 3) verify the candidate genes in progestogenic pathways. This study would improve the understanding of molecular functions by targeting specific pathways in the progestogenic networks, which might open new horizons into the investigation of the normal physiology of the ovaries.

## Methods

### Data acquisition and analysis

Data were acquired from the Gene Expression Omnibus (GEO) (http://www.ncbi.nlm.nih.gov/geo/) database. Two data sets, GSE54584 (this research clarified genetic alteration induced by the inhibition of cyclooxygenase or progesterone receptor) and GSE41836 (This study provided comprehensive information about regulated genes during late follicle development and ovulation induction), were contributed by Kenjiro et al. [17] and Cansu et al. [18], respectively. GSE41836 total contains 12 samples of cumulus oocyte complexes, among which only three samples with human Chorionic Gonadotropins (hCG) treatment were selected. GSE54584 total contains 18 samples, six samples including three samples with hCG treatment and three samples with RU486 (100 mg/kg) treatment in preovulatory were selected [17, 18]. These selected samples were analyzed by the GEO2R (http://www.ncbi.nlm.nih.gov/geo/geo2r/) with respect to the control groups (samples with same dose normal saline treatment). Multiple testing was corrected using the Benjamini-Hochberg procedure to obtain the adjusted *P*-value (*P < 0.05*). Fold changes in the expression of individual genes were calculated and the genes with absolute log_2_ FC > 1 were considered as differentially expressed genes (DEGs). Accordingly, DEGs determined from the selected data sets were identified as DEG41836 and DEG54584, the intersection of which was referred to as the overlapping DEGs.

### Protein-protein interaction (PPI) network analysis

The Search Tool for the Retrieval of Interacting Genes (STRING, version 9.1) database (http://www.bork.embl-heidelberg.de/STRING/) is a pre-computed global resource biological database that includes comprehensive known and predicted protein interaction information. In this study, the STRING 9.1 online tool was used to screen the overlapping DEGs with a confidence score over 0.4. The overlapping DEGs with hCG treatment were collected, then the relevant PPI network was constructed and visualized by Cytoscape software (version 3.0; http://cytoscape.org/) and was further analyzed by ClusterONE, a plugin of Cytoscape. Given that most of biological networks were scale-free, the hub genes were then selected with a connectivity more than 4.

### Function and pathway enrichment analysis

As a comprehensive set of functional annotation tools, the Database for Annotation, Visualization and Integrated Discovery (DAVID) has been used for the systematic and integrative analysis of large gene lists [19]. GO terms are significantly overrepresented in a set of genes from three aspects, including the cellular component, molecular function, and biological process [13, 20]. In this study, the significant GO terms and Kyoto Encyclopaedia of Genes and Genomes (KEGG, http://www.genome.jp/) pathway enrichment analyses of the identified overlapping DEGs were performed using DAVID with the thresholds of *P*-value < 0.05. Results were analyzed by using the IBM SPSS 19.0 software (International Business Machines Corporation, USA).

### Network construction

A biological network was generated by the progesterone-mediated oocyte maturation pathway identified in KEGG. Based on preliminary relationships among differential genes, DEGs from the different treatment groups were assembled. The network analysis identified gene modules of the interconnected genes expressed co-ordinately in target pathways, followed by observation of genes that appeared to be involved in ovulation. The prediction of gene networks which were important to the ovulation process were validated using hCG- and RU486-stimulated experimental data (GSE54584). Details of the pathways, including constituent reactions, involved complexes, and network relationships, were elucidated in KEGG [20]. We recognized these pathways as elementary reactions that contained one reactant along with its resultant product to generate a gene network, regardless of small molecules.

### Q-PCR verification

Sexually mature female rats (8-week-old, Sprague-Dawley) from Chongqing Medical University were used for the experimental verification. Rats were raised in controlled room conditions of constant temperature (22–25 °C) and 12 h light/dark cycles with adlibitum access to food and water. All experiments were performed according to the guidelines of the National Institutes of Health, and the experimental protocol was approved by the animal-experiment committee.

Seleceted genes were validated through analysis. There were two treatment groups (6 Sprague-Dawley female rats per group for one experiment). The first group was intraperitoneally injected with Pregnant Mare Serum Gonadotropin (PMSG, 20 IU), and then 48 h later, hCG (20 IU) was administered through intraperitoneal injection. The second group was based on the procedures of the first group. Specifically, the second group of rats was gavaged with RU486 (100 mg/kg), a progesterone receptor antagonist, at 37 h post-PMSG. And then, hCG was administered through intraperitoneal injection. Rats were sacrificed and cumulus oocyte complex (COCs) samples were collected after hCG treatment 12 h. Total RNA was acquired from ovary COCs using the extraction/isolation kit (BioTeke, Beijing, China). The RNA samples were reverse-transcribed into complementary DNA with the RevertAid First Strand cDNA Synthesis Kit (Takara, Dalian, China).

Each PCR reaction of q-PCR consisted 2 μl cDNA products, 7.5 μl of the Power SYBR Green PCR Master Mix (Takara, Dalian, China), 4.5 μl of water, and 1 μl of gene-specific primers. q-PCR was performed in the following conditions: activation at 95 °C for 30 s, followed by 40 cycles of 5 s at 95 °C and 30 s at 60 °C. The final melting curve was added to examine the amplification quality, whereas expression of mRNA for β-actin was viewed as an internal control standard. The sequences of the primers from National Center for Biotechnology Information reference sequences are shown in Table 1. The q-PCR results were analyzed using the Origin 8.0 software (Originlab Corporation, MA, US).

**Table 1:** Primer of prediction genes

Gene name(symbol)	Primer sequences	Products size	Gene ID
Progesterone receptor (*PGR*)	F:5’-GGTGGGCCTTCCTAACGAG-3’	119 bp	ID:5241
	R:5’-GACCACATCAGGCTCAATGCT-3’		
Reelin (*RELN*)	F:5’-CGCCTTTGGATTCGGGATCA-3’	126 bp	ID:24718
	R:5’-ATTCACACAGCCTGTGCCAG-3’		
G protein subunit alpha i1 (*GNAI1*)	F:5’-ACTTTTCGTGCTTGCTGGGG-3′	128 bp	ID:25686
	R:5’-CCGGGATCTGTTGAAGCAGG-3′		
Protein kinase C, alpha (*PRKCA*)	F:5’-CTGGGGAAGGGGAGTTTTGG-3′	151 bp	ID:24680
	R:5’-CTTCTCCACCATGGTGCACT-3′		
Caveolin 1 (*CAV1*)	F:’-CGCGCACACCAAGGAGATTG-3’	143 bp	ID:25404
	R:5’-TGCCGTCGAAACTGTGTGTC-3’		
Epidermal growth factor receptor (*EGFR*)	F:5’-ATCCTGATTGGTGCTGTGCG-3’	114 bp	ID:24329
	R:5’-CAACTGCTCGGATGGCTCTG-3’		
Ras homolog family member A (*RHOA*)	F:5’-CAGCAAGGACCAGTTCCCAG-3’	137 bp	ID:117273
	R:5’-CGCAGGCGGTCATAATCTTC-3’		
Zyxin (*ZYX*)	F:5’-TTCCAAGTTCAGCCCTGGTG-3’	148 bp	ID:114636
	R:5’-GGGCTTCTCCTTCTGCTGAG-3’		
Vinculin (*VCL*)	F:5’-AACAGCAGACCTGCCAAAGC-3’	118 bp	ID:305679
	R:5’-CAGCCACAAGTCCACGGATG-3’		
Growth factor receptor bound protein (*GRB*)	F:5’-AATCCCCAGAGCCAAGGCAG-3’	145 bp	ID: 84427
	R:5’-TCCAAACTTGACGGACAGGG-3’		
RAS-relatedprotein-1a (*RAP1A*)	F:5’-TGGGGAAGTCTGCTCTGACA-3’	144 bp	ID:295347
	R:5’-GGATCTCCAGCATGCACTGT-3’		
*β-actin*	F:5’-AAAGACCTGTACGCCAACAC-3’	137 bp	ID: 81822
	R:5’-GTCATACTCCTGCTTGCTGAT-3’		

*F* Forward, *R* Reverse

### Statistical analysis

The statistical significance of change in the gene expression was determined using the Student’s t-tests and posthoc Tukey’s test. The data were shown as a fold change (mean ± S.D) of values of at least three independent experiments and *P < 0.05* was considered statistically significant.

## Results

### Functional and pathway analysis

To identify the genes and pathways involved in progestogenic networks, two data sets (GSE41836 and GSE54584, hCG treatment samples) were subjected to intercommunity analysis using bioinformatics approaches. Total 546 common DEGs were obtained from the overlapping differential genes (*P < 0.05*, absolute log_2_ FC > 1), which were then used for PPI analysis and visualization (Additional file 1: Figure S1). Results from ClusterONE analysis identified four hub genes, including *PGR*, reelin (*RELN*), phosphodiesterase 10A (*PDE10A*) and phospholipase A2 group IVA (*PLA2G4A*) (Additional file 2: Table S1).

In order to further determine the key genes in progestogenic networks, functional and pathway enrichment analyses of these four candidate genes showed that PGR was involved in the 51 functions mainly related to ovulation, such as regulation of the ovulation cycle and steroid hormone receptor activity, and participated the signaling pathway of progesterone regulation follicle maturation. RELN was involved in 47 different functions, such as the activity of protein kinases, and PDE10A was mainly reported in the 20 functions related to the nucleotide binding domain, particularly playing a role in the purine metabolic signaling pathway. Finally, PLA2G4A participated cell proliferation regulation, metabolism and other aspects of the functions, and mainly related to the MAPK signaling pathway and the GnRH signaling pathway (Table 2) [21, 22].

**Table 2:** Information of ovulation candidate genes

Name/symbol gene	Gene description	Functional description	Function number of gene	Pathway number of gene
PGR	progesterone receptor	Process of ovulation	51	1
RELN	reelin	factors binding	47	2
PDE10A	phosphodiesterase 10A	Process of metabolic	20	1
PLA2G4A	phospholipase A2, group IVA (cytosolic, calcium-dependent)	positive regulation of factors activity	11	2

**Fig. 1:**
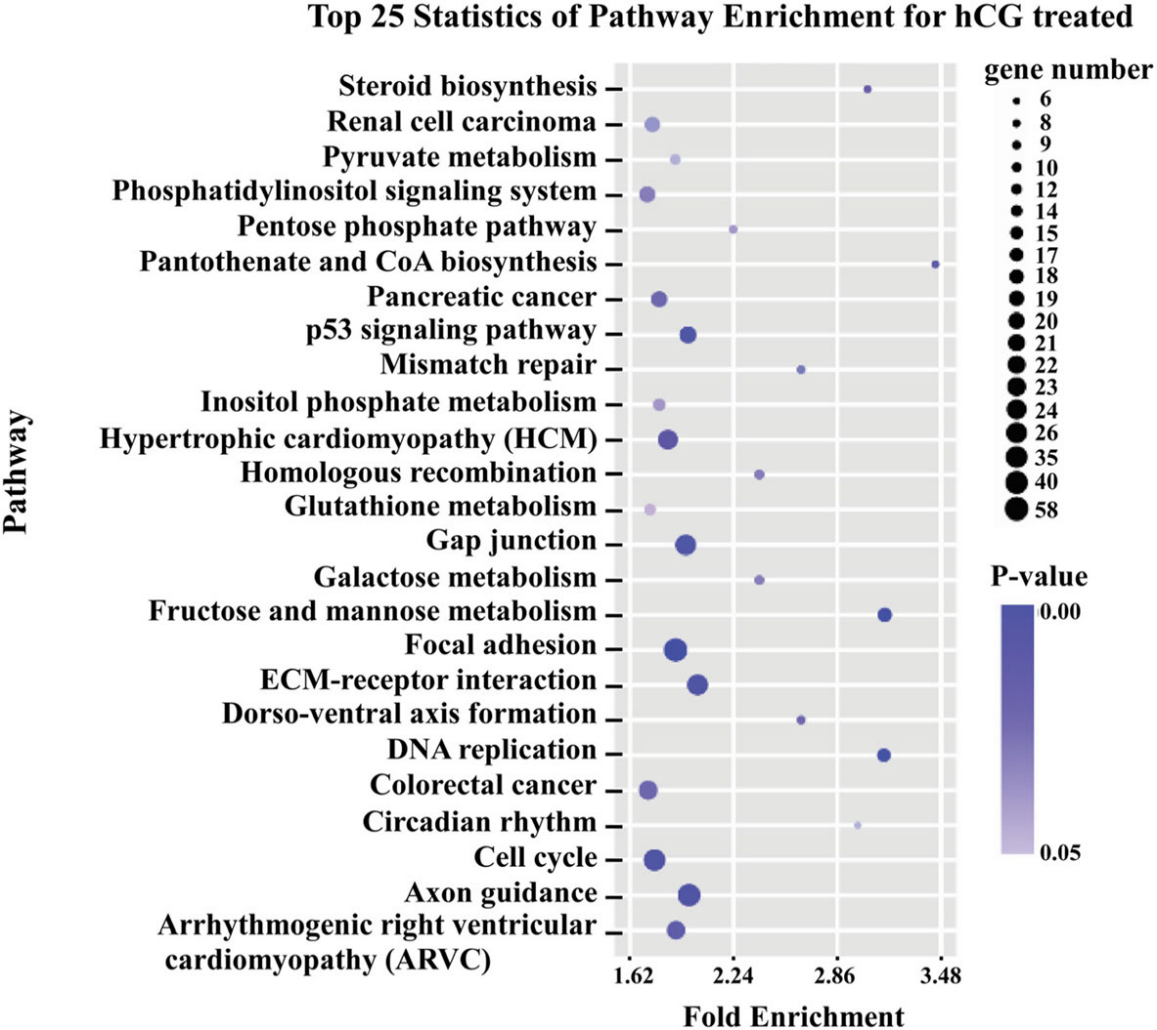
Pathway enrichment analysis. The top 25 statistics pathways identified by pathway enrichment analysis of the 546 DEGs, considering the involved gene numbers and *P*-vaule

Furthermore, we selected the prominent functions of four genes shown in Table 3, including the significant biological process, cellular component, and molecular function related to hCG treatment. In addition, the top 25 statistical pathways were also exhibited as the pathway enrichment analysis results of the 546 DEGs, considering the involved gene numbers and *P*-vaule (Fig. 1).

**Table 3:** The prominent functions enrichment analysis including the molecular function, biological process, and cellular component

Term	*P*-Value	Percent
Molecular function		
Nucleotide binding	1.83E-09	14.41%
Purine nucleotide binding	5.26E-08	12.33%
Purine ribonucleotide binding	5.64E-07	11.67%
Ribonucleotide binding	5.64E-07	11.67%
Adenyl nucleotide binding	6.34E-07	10.17%
Nucleoside binding	1.19E-06	10.24%
Purine nucleoside binding	1.33E-06	10.17%
Ion binding	1.52E-06	23.23%
Cation binding	2.29E-06	22.92%
Metal ion binding	4.40E-06	22.64%
Adenyl ribonucleotide binding	6.25E-06	9.51%
Magnesium ion binding	7.90E-04	2.95%
Guanyl ribonucleotide binding	0.002959769	2.57%
Guanyl nucleotide binding	0.002959769	0.31%
AMP binding	0.005264347	0.66%
Biological process		
Multicellular organism reproduction	0.0001	3.13%
Reproductive process in a multicellular organism	0.0001	3.13%
Morphogenesis of a branching structure	0.0001	1.25%
Tissue morphogenesis	0.0001	2.01%
Epithelium development	0.0001	2.22%
Tube morphogenesis	0.0001	1.56%
Development of primary sexual characteristics	0.0001	1.08%
Sexual reproduction	0.0001	2.92%
Sex differentiation	0.0002	1.25%
Gamete generation	0.0004	2.50%
Branching morphogenesis of a tube	0.0014	0.90%
Ovulation cycle process	0.0021	0.56%
Ovulation cycle	0.0026	0.56%
Female pregnancy	0.0075	0.66%
Steroid hormone receptor signaling pathway	0.0251	0.28%
Phospholipid metabolic process	0.0472	1.15%
Phosphorus metabolic process	0.000018103	5.94%
Cellular component		
Vesicle	0.023864658	3.16%
Cytoplasmic vesicle	0.026121829	3.09%
Extracellular matrix	2.72E-06	2.60%
Extracellular region part	7.54E-06	5.35%
Proteinaceous extracellular matrix	9.05E-06	2.47%
Cell projection	7.94E-06	3.82%
Extracellular space	0.008741592	3.23%
Neuron projection	0.011946122	1.70%

### Pathway regulation and gene network analysis

Progestogenic pathways are a coordinated process initiated by hormones that activate ovulation. PGR and RELN play an important role in this process. PGR is an upstream regulator of the progestogenic pathways in ovulation [23]. RELN acts as a factor promoting the end of follicle growth [24]. To verify whether the genes and networks of progestogenic pathways had potential theoretical significance, *PGR* and *RELN* were analyzed to investigate the effects of differential expression in this process.

**Fig. 2:**
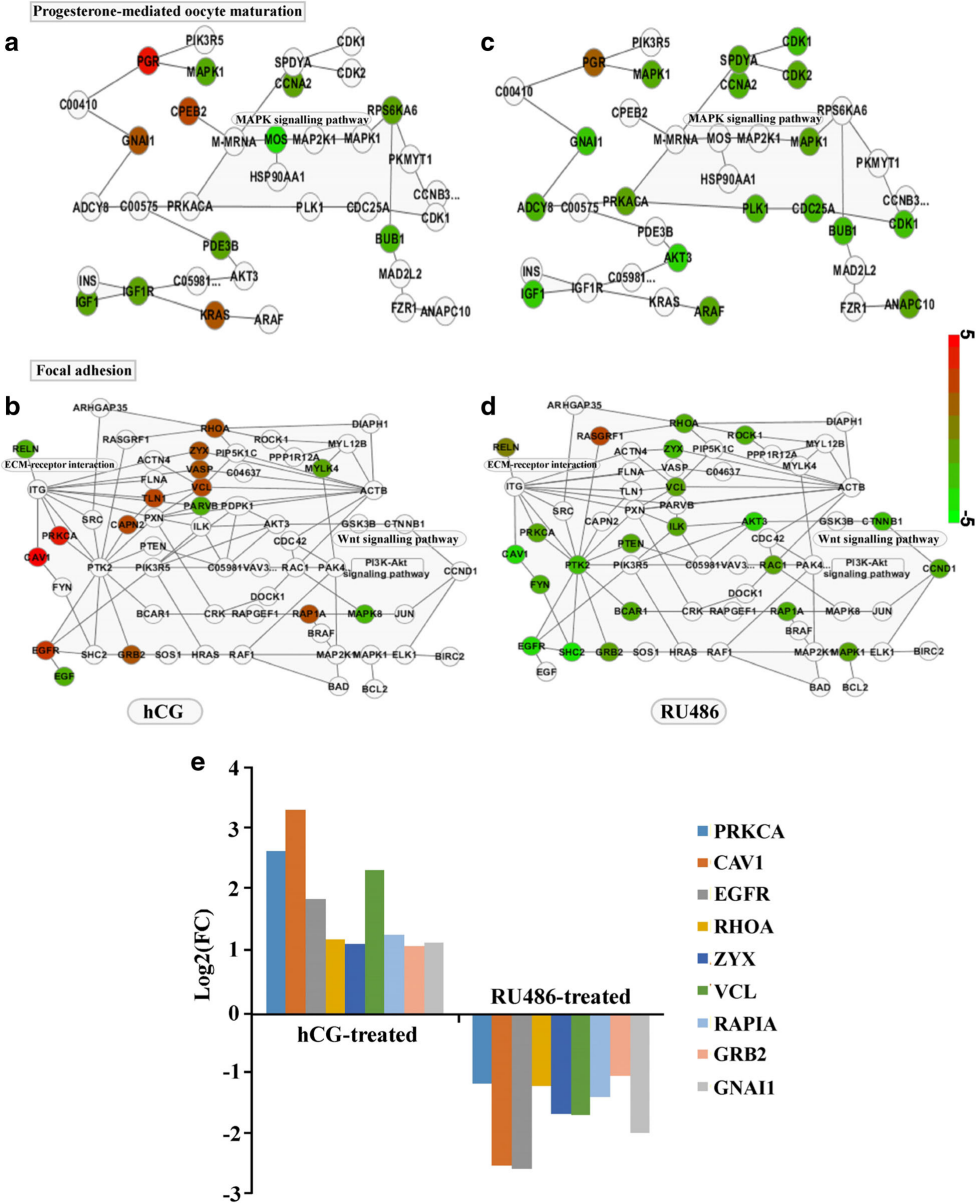
Gene expression analysis with hCG and RU treatment in preovulatory follicles from GSE54584. All validated genes were significantly differentially expressed in hCG- and RU486-treated ovarian. **a** and **c** were hCG-treated group, and **b** and **d** were RU486-treated group. Nodes in red represent the genes with expression level above the mean and nodes in green represent the genes with expression below the mean. The intensity of the pseudo-color reflects cross parts. **e**, Differentially expressed genes showed by visualized analysis with *p*-value < 0.05 and absolute log_2_ FC > 1

To characterize genes’ alterations in preovulatory processes, gene expression data were analyzed related to hCG and RU486-treated experiments (GSE54584 data). Results demonstrated that significant changes in gene expression were observed comparing with the control groups. However, expression profiles of *PGR* in hCG and RU486-treated groups were significantly different. This transcript is known to be involved in progesterone-mediated oocyte maturation pathways and to interact with *GNAI1*, *CPEB2* and *KRAS* (Fig. 2a). Four transcripts (*MAPK1*, *CCNA2*, *IGF1* and *BUB1*) were significantly down-regulated in the two treatments (Fig. 2a and c). However, unlike the signaling pathways involving *PGR*, hCG and RU486-treated produced differential changes in *RELN* in follicular growth or focal adhesion pathways. Up-regulated transcripts in hCG-treated included *PRKCA* (> 5-fold), *CAV1* (> 6-fold), *EGFR* (> 3-fold), *RHOA* (> 2-fold), ZYX (> 2-fold), *VCL* (> 4-fold), *RAP1A* (> 2-fold), and *GRB2* (> 2-fold). In contrast, these transcripts were down-regulated in RU486-treated (Fig. 2b, d and e). In addition, some genes (*MAPK1*, *BUB1*, *CANA2* and IGF1) involved in the RELN pathway were down-regulated in both hCG and RU486 treatment (Additional file 3: Table S2). These expression differences were considered relevant to the induction of ruptured and un-ruptured follicles and other features such as inflammation, tissue remodeling, extracellular matrix release, and steroid metabolism [2, 7, 17].

**Fig. 3:**
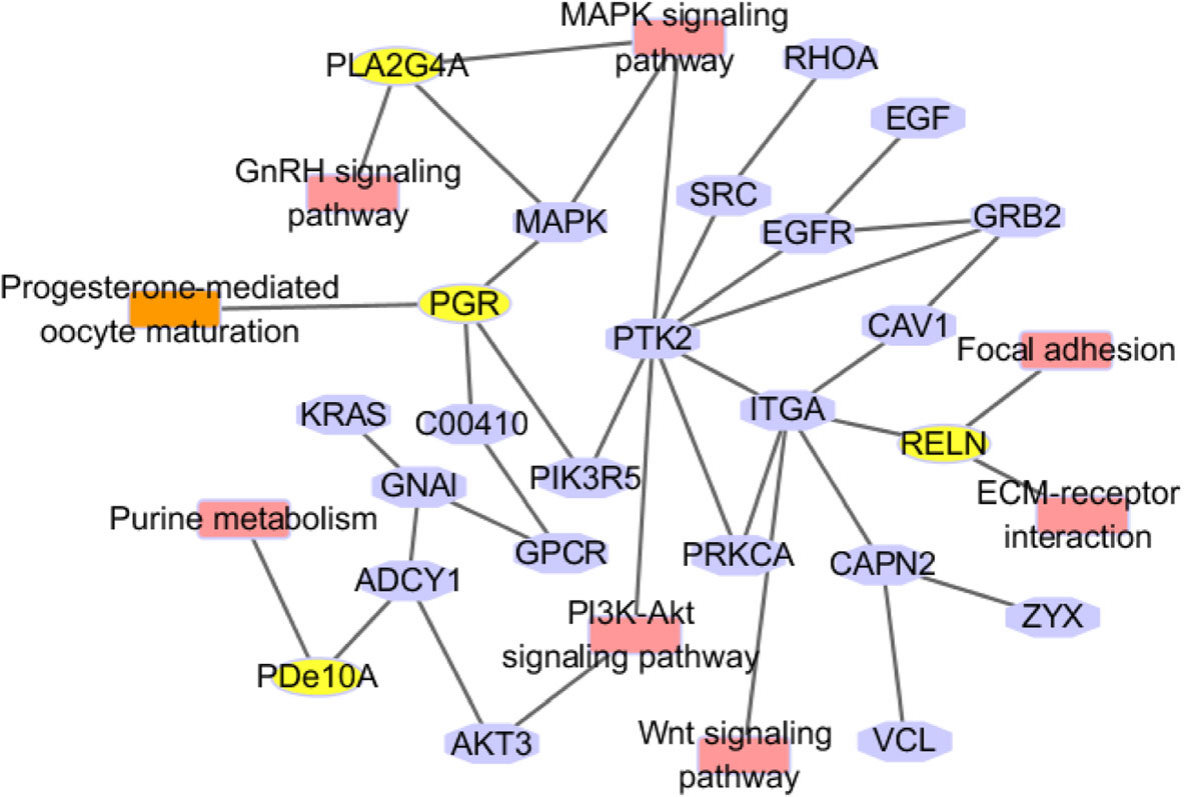
Visualized progesterone-related genes network. Nodes in different colors represent different target genes or pathways. Purple sexangles represent node genes (*MAPK*, *SRC*, *PTK2*, *ITGA*, *CAPN2*, *GPCR*, *ADCY1*) and predicted target genes (*GNAI1*, *PRKCA*, *CAV1*, *EGFR*, *RHOA*, ZYX, *VCL*, *GRB2* and *RAP1A*) in this study; yellow color ellipses represent genes between networks; and red color rectangles reflect pathways involved in progestogenic networks

New gene network construction of the progestogenic pathway was based on primary network relationships between the four genes and pathways of PGR and RELN. The network combined 8 pathways (progesterone-mediated oocyte maturation, MAPK signaling pathway, GnRH signaling pathway, purine metabolism, PI3K-Akt signaling pathway, Wnt signaling pathway, ECM-receptor interaction, and focal adhesion) with node genes *PIK3R5*, *MAPK,* and *ADCY1*, then added nine predicted genes (*GNAI1*, *PRKCA*, *RHOA*, ZYX, *VCL*, *RAP1A*, *CAV1*, *EGFR* and *GRB2*) to the network. A combined method was taken, regardless of small molecules or undetected genes (Fig. 3).

### Validation of predicted results by q-PCR

**Fig. 4:**
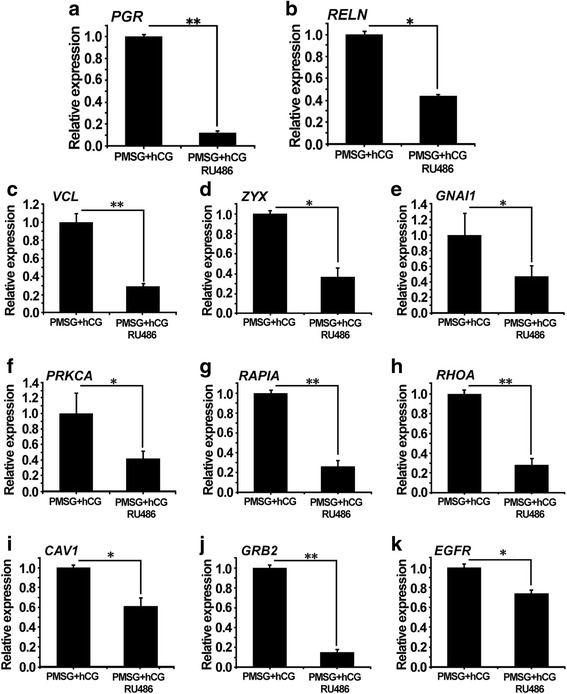
Validation of progesterone-related predicted genes by q-PCR analysis. The two groups of rats were treated by PMSG and hCG or PMSG, hCG and RU486. Total RNA of COCs was extracted for q-PCR analysis of the genes expression. **a**-**b**: *PGR* and *RELN* expression; **c**-**k**: expression of the nine genes (*GNAI1*, *PRKCA*, *CAV1*, *EGFR*, *RHOA*, ZYX, *VCL*, *GRB2* and *RAP1A*, respectively). Different asterisks indicate statistically significant differences (*, *P* < 0.05; **, *P* < 0.01). Error bars represent S.D.

In order to verify whether the differential genes (*GNAI1*, *PRKCA*, *RHOA*, ZYX, *VCL*, *RAP1A*, *CAV1*, *EGFR* and *GRB2*) in the progesterone signaling pathway were indeed regulated by PGR, they were detected by q-PCR. This was the quantitative detection of samples from hCG- and RU486-treated rats after the extraction of COCs. The results of q-PCR showed that the expression of PGR was almost completely inhibited after RU486 treatment, and the expression of RELN was also decreased (Fig. 4a and b). In addition, it was found that the nine predicted genes (*GNAI1*, *PRKCA*, *RHOA*, ZYX, *VCL*, *RAP1A*, *CAV1*, *EGFR* and *GRB2*) exhibited a differential change (Fig. 4c-k). The expression of *VCL*, *RHOA*, *RAP1A* and *GRB2* decreased by about 3-fold compared with the control group, and the significant decreases of ZYX, *GNAI1*, *CAV1* and *PRKCA* were about 2-fold. The change of *EGFR* was inhibited, although the trend became below par. This may also be subject to the regulation of other factors in the process of ovulation.

## Discussion

One of the challenges in mammalian ovulation research is to investigate the basic regulatory and physiological mechanisms [25]. The present study has identified genes with special importance in unique developmental processes. Selectively GSE41836 and GSE54584 data served as accessible data sets for studying the progestogenic-related gene networks in mammals [10]. Herein, we highlighted ovarian physiology and identified many new progestogenic-related genes in this study.

This study also identified several novel initiation factors of ovulation. Regulations of hormones and steroidogenesis are regarded as an initiation process, which provides detailed fundamental information on the profound changes regulated by gene networks in ovulation. Hormone (FSH and LH) induced primary follicle maturation and eventually approached the pre-ovulatory stage in mice and rats [2, 26]. Pituitary-released LH forms the LH surge which subsequently initiates the estrous cycle in rodents in response to the high levels of estrogen [27]. The steroid hormone progesterone, regulated by the LH surge, plays a key role in ovulation [28]. The effects of progesterone were mediated by PGR, and its expression was observed in the granulosa cells of pre-ovulatory follicles following hCG treatment in mice and rats [29]. PGR was accompanied by the coordinated expression of progesterone biosynthetic enzymes, including steroidogenic acute regulatory protein (Star) and cholesterol side-chain cleavage enzyme (Cyp11a1) [30, 31]. An expression increase of Star and Cyp11a1 was observed following hCG stimulation, but a decrease was in RU486-treated mice, which revealed a pattern of expression related to the progestogenic pathway.

In the progestogenic pathway, PGR expression is induced primarily in granulosa cells of the pre-ovulatory follicles [2]. PGR is a transcription factor involved in multiple processes including oocyte maturation, ovulation, ovulation cycle process, epithelial cell development, and steroid hormone receptor signaling pathways. PGR directly mediates ovulation and indirectly regulates other ovulation-related factors. The expression of *RELN*, a regulator of follicle growth factor like IGF2 [24], decreased in hCG-treated rats (Fig. 2c). It binded with the receptor LRP8 to regulate follicular growth in cattle. MAPK8IP1 encoding LRP8 intracellular interacting partner was also expressed in granulosa cells of the dominant follicle. This differential expression pattern suggested an RELN/LRP8/MAPK8IP1 paracrine interaction to regulate follicular growth until ovulation [32]. *PDE10A*, one of the PDE families, is also expressed in bovine cumulus cells by cAMP-PDE activity [33]. Mammalian oocytes initiate meiosis in the foetus but remain arrested at the diplotene stage of the first meiotic prophase, before being potentiality to resume by the ovulatory luteinising hormone pulse [34]. High levels of cAMP keep oocytes in the meiotic arrest phase by activating cAMP-dependent protein kinase. As for *PDE10A*, effects of ovulation on the process are uncertain. Functional and pathway analysis found that *PLA2G4A* was involved in the MAPK and GnRH signaling pathways. Many studies have confirmed that ovulation is associated with an LH/hCG-dependent induction of *PLA2G4A* in granulosa cells via the adenylyl cyclase/cAMP pathway [35]. Kurusu et al. [36] found that *PLA2G4A* optimizes ovulation and fertilization when coupled with prostaglandin endoperoxide synthase in female mice lacking *PLA2G4A*. This study found that four genes (*PGR*, *RELN*, *PDE10A*, and *PLA2G4A*) developed an expression pattern consistent with a progestogenic pathway during ovulation in mammals. This analysis suggests that these four genes can form a network of cellular pathways or nodes to regulate ovulation.

Progestogenic pathway plays an important role in the “story” of follicle maturation and ovulation. In order to explore extensions of the progestogenic pathway, this study investigated the four genes that were involved in this pathway. An analysis of the four genes from the KEGG pathway and PPI showed that *MAPK*, *PIK3R5*, *PTK2*, *ITGA*, and *ADCY1* had the most regulatory connections to adjacent genes (Fig. 4). These genes are considered important in regulating features involved in the progestogenic network or as downstream factors of the network. Genes involved in each pathway may act in concert with accomplishing distinct processes during ovulation including prostaglandin synthesis (*Ptgs2* and *PLA2G4A*) [7], cholesterol uptake (*Ldlr* and *Scarb1*) [37], regulation of progesterone synthesis (*Star*) [30], inactivation of estrogen (*Sult1e1*) [38], and downstream effectors of LH signaling (*Pgr*, *Cebpb*, *Areg*, *Ereg*, and *Adamts1*) [6, 26, 29, 39]. These processes regulate inflammation, follicle-wall degradation, hormone balance and the induction of certain paracrine factors. In addition, genes encode proteins involved in various biology process, including reproductive developmental processes and granulosa cell expansion (*Tgfb2*, *Foxl2*, *Pde3a*, *Esr2*, *Fshr*, *Ccnd2*) [40], amino acid degradation and ketogenesis (*Hmgcs2*, *Cpt1b*) [41]. These results presented candidate genes involved in independent cellular pathways to carry out relevant functions in progestogenic pathways.

Interestingly, when the rats were subjected to hCG and RU486 treatment, the results indicated that most of the genes involved in PGR and RELN pathways were differentially regulated. Furthermore, these results illustrated that focal adhesion, EMC-receptor interaction and MAPK signaling pathways were affected in hCG- and RU486-treated rats. MAPK signaling pathways provoke ovulation and collapse of the follicle, which were stimulated by a variety of exotic signals (MAP kinases 1 and 3, extracellular-regulated protein kinases 1 and 2 (ERK1/2)) [42]; the Wnt-signaling and PI3K-Akt signaling pathways were not significantly affected by hCG treatment (Fig. 2c). Conti and Makker [43, 44] found that the PI3K/PTEN/Akt signaling pathway and cAMP/ERK/EGF network induced oocyte and meiotic maturation. It is noteworthy that the PDE family participated in the cAMP pathway and research suggests that it also regulates ovulation. Sasseville et al. [33] indicated the establishment of a model of cell-specific expression of PDEs in the ovarian follicle. This suggested that *Pde3a* was a major isoform of oocyte PDE, *Pde4d* was the major granulosa cell PDE isoforms and *PDE10A* was expressed in cumulus cells. Cell-specific expression of cAMP-PDEs in ovarian follicles has implications for understanding the hormonal regulation of folliculogenesis [45]. Further studies are needed to determine how Pde10a-Adcy1 participates in progestogenic pathways. Pathway analysis and gene validation indicated that *RELN* could participate in PI3K-Akt signaling by PTEN, but the molecular mechanism of this process remained unclear. However, when *RELN* combines with receptor LRP8 to regulate follicular growth, the pattern of LRP8 expression correlates with the capacity of follicles to synthesize higher steroid levels in cattle [32]. Subsequently, the phase of follicular development, including the LH surge, changes the relationship between progestin and androgen. Enzymes required for progestin include hydroxy-delta-5-steroid dehydrogenase, 3 beta- and steroid delta-isomerase (Hsd3b2), and Cyp11a1. This suggests that RELN and LRP8 may be regulated in progestogenic pathways, but human studies have not verified this regulation. Pathway validation indicated the *PRKCA* exhibited significant changes in RELN regulation (Fig. 2b and d). This validation also connected *Ptk2* to progesterone-mediated oocyte maturation which confirmed the relationship between RELN and PGR. Details of this interaction require further investigation. Relative expression results using q-PCR also found that *GNAI1* was considered as a PGR indirect regulatory factor, but this process was not previously associated with ovulation. *GNAI1*, *PRKCA*, *RHOA*, ZYX, *VCL*, *RAP1A*, *CAV1*, *EGFR* and *GRB2* produced changes in progestogenic pathways and these factors were considered as ovulation-associated factors. *PLA2G4A* is involved in the progestogenic pathway by paracrine regulation, which plays an important role in ovulation. The ovulatory surge of LH regulates the expression of each component of the PGE_2_ synthesis-metabolism-transport pathway to mediate key ovulatory events including cumulus expansion, rupture and release of oocytes [7].

## Conclusions

In sum, a systematic bioinformatics analysis approach could provide an overview of a particular developmental process. Results suggested that the four genes (*PGR*, *RELN*, *PDE10A* and *PLA2G4A*) were involved in novel progestogenic networks (Fig. 3). These networks provided important regulatory genes and signaling pathways which were involved in ovulation. At the same time, hCG- and RU486-treated experiments confirmed that candidate genes (*GNAI1*, *PRKCA*, *RHOA*, ZYX, *VCL*, *RAP1A*, *CAV1*, *EGFR* and *GRB2*) were involved in ovulation.

This study has shown that the above genes are selectively or highly differentially expressed in progestogenic pathways, which provides important information for future downstream analysis to identify the significant genes in ovulation and develop effective strategies for fertility control.

## Additional files

Additional file 1: **Figure S1.** The significant modules in the protein-protein interaction network with MCODE. Nodes denoted proteins (genes). In specific, the yellow denoted seed proteins; the red denoted the up-regulated proteins; and the green denoted down-regulated proteins; the width of edges was determined according to the combined score of the protein-protein interaction relationships. Abbreviation: MCODE, Molecular Complex Detection. (DOCX 115 kb)

Additional file 2: **Table S1.** Four genes information from analysis of Cluster One. (DOCX 13 kb)

Additional file 3: **Table S2.** The list of significantly differentially expressed genes of the pathways involving RELN and PGR. (DOCX 14 kb)

## Abbreviations

Cyp11a1: Cholesterol side-chain cleavage enzyme
E2: Estradiol
ECG: Equine chorionic gonadotrophin
FSH: Follicle-stimulating hormone
GnRH: Gonadotropin-releasing hormone
GO: Gene Ontology
HCG: Human chorionic gonadotrophin
Hsd3b2: Hydroxy-delta-5-steroid dehydrogenase, 3 beta- and steroid delta-isomerase
Il6: Interleukin 6
LH: Luteinizing hormone
OCC: Oocyte cumulus complex
PDE10A: Phosphodiesterase 10A
PGR: Progesterone receptor
PLA2G4A: Phospholipase A2 group IVA
Ptgs2: Prostaglandin-endoperoxide synthase 2
RELN: Reelin
Vegfa: Vascular endothelial growth factor
