# Supplementary material for: Identification of new progestogen-associated networks in mammalian ovulation using bioinformatics
Source: BMC Syst Biol. 2018 Apr 3;12:36. doi: 10.1186/s12918-018-0577-7 (PMC5883354; doi:10.1186/s12918-018-0577-7)

**Supplementary Figure 1.** The significant modules in the protein-protein interaction network with MCODE. Nodes denoted proteins (genes). In specific, the yellow denoted seed proteins; the red denoted the up-regulated proteins; and the green denoted down-regulated proteins; the width of edges was determined according to the combined score of the protein-protein interaction relationships. Abbreviation: MCODE, Molecular Complex Detection.

**Supplementary Figure 1**


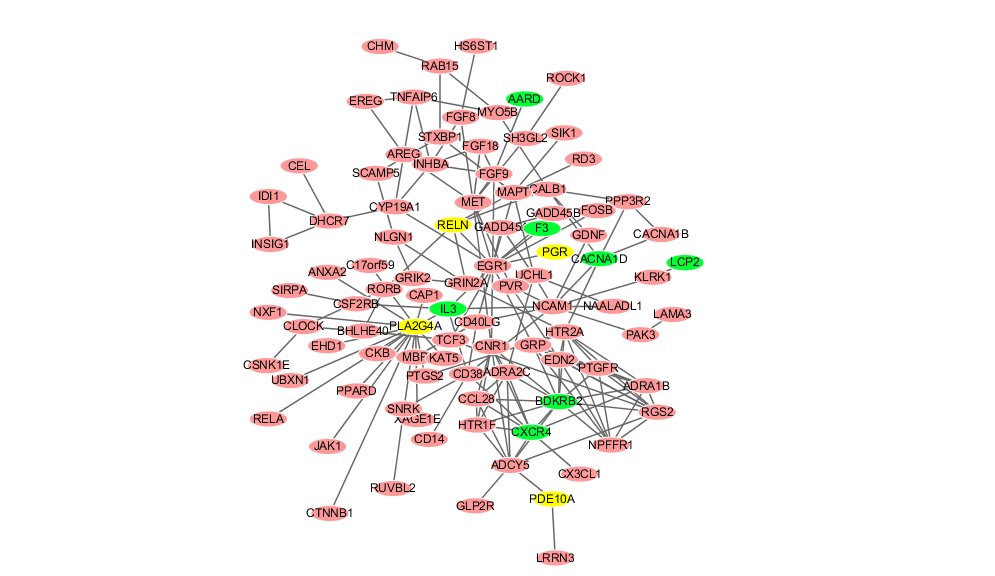

Supplement: Supplementary file 1 — Figure S1. The significant modules in the protein-protein interaction network with MCODE. Nodes denoted proteins (genes). In specific, the yellow denoted seed proteins; the red denoted the up-regulated proteins; and the green denoted down-regulated proteins; the width of edges was determined according to the combined score of the protein-protein interaction relationships. Abbreviation: MCODE, Molecular Complex Detection. (DOCX 115 kb) [file 12918_2018_577_MOESM1_ESM.docx]
