# Supplementary material for: Identification of new progestogen-associated networks in mammalian ovulation using bioinformatics
Source: BMC Syst Biol. 2018 Apr 3;12:36. doi: 10.1186/s12918-018-0577-7 (PMC5883354; doi:10.1186/s12918-018-0577-7)
Supplement: Supplementary file 2 — Table S1. Four genes information from analysis of Cluster One. (DOCX 13 kb) [file 12918_2018_577_MOESM2_ESM.docx]

Table S1. Four genes information from analysis of Cluster One

| Gene name | Nodes | *P*-vaule |
| --- | --- | --- |
| PGR | 9 | 1.915E^-4^ |
| PLA2G4A | 8 | 0.012 |
| RELN | 6 | 0.018 |
| Pde10a | 4 | 0.023 |
