# Supplementary material for: Identification of new progestogen-associated networks in mammalian ovulation using bioinformatics
Source: BMC Syst Biol. 2018 Apr 3;12:36. doi: 10.1186/s12918-018-0577-7 (PMC5883354; doi:10.1186/s12918-018-0577-7)
Supplement: Supplementary file 3 — Table S2. The list of significantly differentially expressed genes of the pathways involving RELN and PGR. (DOCX 14 kb) [file 12918_2018_577_MOESM3_ESM.docx]

Table S2. The list of significantly differentially expressed genes of the pathways involving RELN and PGR

| Gene title | Gene name | hCG-treated  (log_2_ FC) | RU486-treated  (log_2_ FC) |
| --- | --- | --- | --- |
| EGF | epidermal growth factor | -1.3800 |  |
| PARVB | parvin, beta | -1.3500 |  |
| MYLK4 | myosin light chain kinase family, member 4 | -1.0900 |  |
| MAPK8 | mitogen-activated protein kinase 8 | -1.5900 |  |
| FYN | FYN proto-oncogene, Src family tyrosine kinase |  | -1.4588 |
| SHC2 | SHC (Src homology 2 domain containing) transforming protein 2 |  | -2.8216 |
| PTK2 | protein tyrosine kinase 2 |  | -1.7576 |
| PTEN | phosphatase and tensin homolog |  | -1.1949 |
| ILK | integrin-linked kinase |  | -1.1502 |
| ROCK1 | Rho-associated coiled-coil containing protein kinase 1 |  | -1.3622 |
| AKT3 | v-akt murine thymoma viral oncogene homolog 3 |  | -2.3048 |
| RAC1 | ras-related C3 botulinum toxin substrate 1 |  | -1.0798 |
| CTNNB1 | catenin (cadherin associated protein), beta 1 |  | -1.7864 |
| CCND1 | cyclin D1 |  | -1.3335 |
| BCAR1 | breast cancer anti-estrogen resistance 1 |  | -1.2977 |
| CCNA2 | cyclin A2 | -1.0100 | -1.4090193 |
| IGF1 | insulin-like growth factor 1 | -1.3800 | -1.8183469 |
| BUB1 | BUB1 mitotic checkpoint serine/threonine kinase | -1.1400 | -1.7490666 |
| MAPK1 | mitogen activated protein kinase 1 | -1.3300 | -1.2099136 |
